# Comparison method for community detection on brain networks from neuroimaging data

**DOI:** 10.1007/s41109-016-0007-y

**Published:** 2016-08-16

**Authors:** Fumihiko Taya, Joshua de Souza, Nitish V. Thakor, Anastasios Bezerianos

**Affiliations:** 1 Singapore Institute for Neurotechnology (SINAPSE), Centre for Life Sciences, National University of Singapore, 28 Medical Drive, #05-Cor, 117456 Singapore, Singapore; 2 Institute of High Performance Computing (IHPC), Agency for Science, Technology and Research (A*STAR), Singapore, Singapore; 3 Department of Electrical & Computer Engineering, National University of Singapore, Singapore, Singapore; 4 Department of Biomedical Engineering, National University of Singapore, Singapore, Singapore; 5 Department of Biomedical Engineering, Johns Hopkins University, Baltimore, MD USA; 6 School of Medicine, University of Patras, Patras, Greece

**Keywords:** Brain network, Community detection, “Ground truth”, Permutation test, Normalized Mutual Information (NMI), Multiple-subject data, Functional Magnetic Resonance Imaging (fMRI), Brain atlases

## Abstract

The brain is a complex system consisting of regions dedicated to different brain functions, and higher cognitive functions are realized via information flow between distant brain areas communicating with each other. As such, it is natural to shift towards brain network analysis from mapping of brain functions, for deeper understanding of the brain system. The graph theoretical network metrics measure global or local properties of network topology, but they do not provide any information about the intermediate scale of the network. Community structure analysis is a useful approach to investigate the mesoscale organization of brain network. However, the community detection schemes are yet to be established.

In this paper, we propose a method to compare different community detection schemes for neuroimaging data from multiple subjects. To the best of our knowledge, our method is the first attempt to evaluate community detection from multiple-subject data without “ground truth” community and any assumptions about the original network features. To show its feasibility, three community detection algorithms and three different brain atlases were examined using resting-state fMRI functional networks. As it is crucial to find a single group-based community structure as a representative for a group of subjects to allow discussion about brain areas and connections in different conditions on common ground, a number of community detection schemes based on different approaches have been proposed. A non-parametric permutation test on similarity between group-based community structures and individual community structures was used to determine which algorithm or atlas provided the best representative structure of the group. The Normalized Mutual Information (NMI) was computed to measure the similarity between the community structures. We also discuss further issues on community detection using the proposed method.

## Background

Recent development in neuroimaging devices such as magnetic resonance imaging (MRI) and electroencephalography (EEG) enable us to record human brain activity with high temporal and spatial resolutions. As the brain is known as a complex network consisting of distributed brain areas dedicated to different functions, it is natural to shift towards brain network analysis from brain mapping to understand the functional integration in the brain (Friston 2011). Additionally, the development in computational methods has facilitated studies on brain networks using big data. Brain connectome is a promising approach, employing complex network analysis based on graph theory for investigating topological architecture of the brain network (Sporns 2013).

For better understanding of the relationship between brain regions and their topological roles in the brain network, network analysis has been applied to structural and functional brain networks derived from neuroimaging data to quantify the topological characteristics of the network using graph theoretical network metrics such as clustering coefficient, shortest path length, small-worldness, and node centrality (Rubinov and Sporns 2010). Although these metrics provide useful information regarding global and local properties of the brain network, they do not provide any information about the intermediate scale of the network. As such, community detection techniques have been used to investigate the mesoscale network organization (Meunier et al. 2010).

The community structure analysis is one of the most important field of complex networks as networks in nature often show hierarchical, modular organization (Fortunato 2010; Fortunato and Castellano 2012; A. Lancichinetti and Fortunato 2009; M. E. J. Newman 2012). Communities or clusters are usually regarded as subgraphs of a network, with dense connections within the subgraphs, that are weakly linked each other. To identify community structure of networks, a variety of community detection algorithms have been proposed, and have been applied to various networks, e.g., metabolic networks (Guimera and Nunes Amaral 2005), citation networks (Rosvall and Bergstrom 2008), co-authorship network (Luzar et al. 2014), and Twitter network (Beguerisse-Diaz et al. 2014). However, there are no established schemes to perform the community structure analysis on the neuroimaging data. For the neuroimaging data, it is of great importance to find a representative, group-based community structure for a group of subject to have discussions about brain states in different conditions on common ground. Although a number of approaches for finding a consensus cluster have been proposed (Dong et al. 2014; Kumar et al. 2011; Ozdemir et al. 2015; Strehl and Ghosh 2003; Tang et al. 2009), not only the community detection algorithms, but also pre-processing of data and methods for construction of networks influence the results. Additionally, although community detection algorithms are usually evaluated by comparing detected communities with “ground truth” communities, which are often provided independently of topological properties of networks as node metadata (Hric et al. 2014), such “ground truth” community is unknown or is no use for studies on neuroimaging data. Rather than comparing the obtained community with a priori metadata communities, it would be more important to identify a common group-based community structure representative of individual structures. Furthermore, community detection algorithms identify community structure, which are substantially different from metadata groups classified with non-topological features of nodes, particularly for large networks (Hric et al. 2014). As such, comparison methods for community detection schemes independent of “ground truth” community would be required to establish the scheme for community structure analysis on neuroimaging data.

In this study, we propose a method to compare different community structure analysis approaches for multiple-subject data in terms of the agreement of a group-based community structure with individual community structures (de Souza et al. 2015). In the proposed method, a non-parametric permutation test is used to examine which community structure shows a greater agreement with individual structures. The group-based community structure is compared with each individual community structure, and the number of subjects showing greater similarity with the group-based community is examined, instead of averaged values, which has been used for finding the best partition of networks (Strehl and Ghosh 2003) or selecting a representative among individual structures (Meunier et al. 2009b). To quantify similarity between community structures, Normalized Mutual Information (NMI) (Fortunato 2010; Kuncheva and Hadjitodorov 2004) is computed for every pair of the group-based community and individual communities. A lot of measures for comparing different community structures other than NMI have been proposed (Fortunato 2010; Lancichinetti et al. 2009; Meilă 2007), and can be used instead of NMI, depending on the presumptions about the networks and the communities.

To show the feasibility of the method for comparing different community detection approaches, three different community detection algorithms for multiple subjects (i.e., the “virtual-typical-subject” (VTS), the “individual structure” (IS) and the “group analysis” (GA) approaches are compared. In the VTS or consensus averaging approach, we suppose that group averaged data reflect a typical pattern of the data, ignoring inter-subject variability. In the IS approach, a community detection algorithm is applied to each individual network and a single group-based community is extracted from the obtained individual communities. The problem to find a consensus cluster from multiple known clusters without accessing the original features has been known as clustering ensembles or multiple graph clustering, and has been studied intensively (Dong et al. 2014; Kumar et al. 2011; Strehl and Ghosh 2003; Tang et al. 2009). As it is requisite to detect individual communities before obtaining a group-based community, the IS approach is usually computationally expensive. To overcome the problems with VTS and IS approaches, a GA method, in which a common modularity function from multiple subjects is optimized while searching for a common group-based community directly from individual networks, was proposed (Y. Liu et al. 2014). As this approach does not require obtaining individual community structures, it is computationally less expensive than the IS approach. The Liu’s method was tested on simulated EEG networks with predefined “ground truth” community structure, outperforming the VTS approach, and was applied to real EEG data to use the obtained community structure for investigating inter- and intra-cluster information flow (Liu et al. 2014). However, the method is yet to be evaluated with actual neuroimaging data. Ozdemir and colleagues have also proposed a GA-based community detection algorithm and compared it with the VTS approach and several algorithms following the IS approach without “ground truth”, in terms of the agreement between the common community structure and each subject’s connectivity graph on the assumption that rank distribution of inter- and intra-cluster connection strengths are indicative of quality of a cluster (Ozdemir et al. 2015). Differently from their approach, our comparison method examines the agreement of the common community structure with individual community structures without any assumptions about the original network features.

In addition to the algorithms for community detection, node definition in network graphs is of great importance in community structure analysis. For graph theoretical analysis on neuroimaging data, brain networks are usually constructed by dividing whole brain volume into brain regions, which are defined anatomically, functionally or based on connectivity. However, different brain atlases can be mutually inconsistent and produce different results (Stanley et al. 2013). Also, as the number of nodes defined by brain atlases increases, the computational costs for community detection get more expensive. Although voxels can also be regarded as nodes of a brain network graph, it is often too fine for the problem under investigation and computationally too expensive. To show that the proposed method can be employed to compare different brain atlases for parcellation, three brain atlases, which are popular and easily obtainable, were examined.

We demonstrate that the proposed method based on the permutation test and NMI would be a powerful tool for assessing different community detection algorithms and different brain atlases in terms of the agreement of a group-based community structure with individual structures, without “ground truth” metadata and accessing the original network features. Undirected, weighted network graphs derived from resting-state fMRI data were used for the community structure analysis. We would like to emphasize that the goal of this paper is not to find the best scheme for the community structure analysis on neuroimaging data from a vast number of possible combinations of the methods, but to provide a tool to assess different schemes. Although the methods based on the popular Louvian algorithm (Blondel et al. 2008) were used for the all of the approaches examined in this study, the other algorithms such as Informap (Rosvall and Bergstrom 2008) or NetExplorer (Massaro et al. 2012) have been compared for cat and macaque cortical networks (Gronchi et al. 2014) and could outperform the Louvian method for the fMRI functional networks as well.

## Methods

**Fig. 1:**
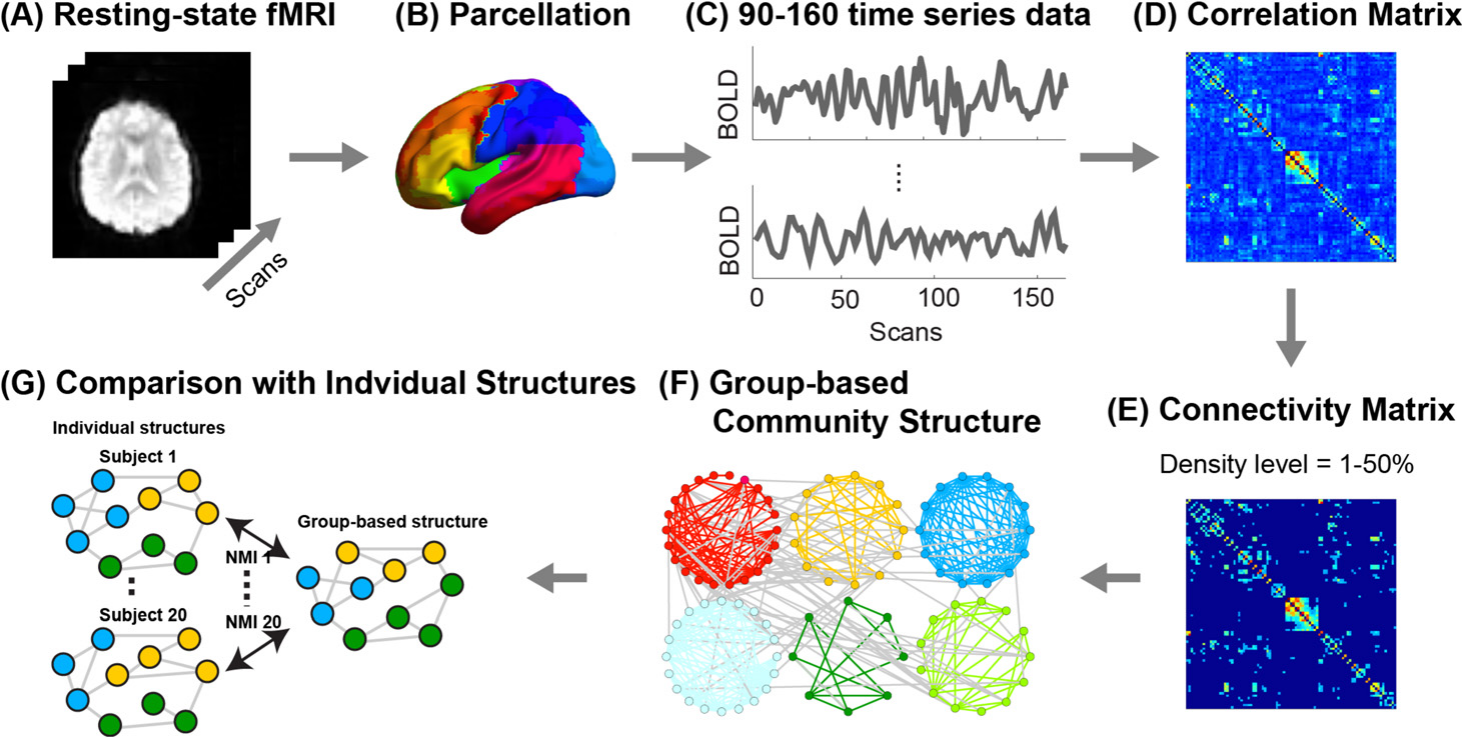
The processing steps of the community detection analysis. For each subject, resting-state fMRI data were acquired (**a**) and parcellated into ROIs with a brain atlas (AAL, HOA or Dosenbach atlas) (**b**) to get time-series data of brain activity (**c**). A correlation matrix was obtained from the time-series data (**d**) and a network graph was represented by a connectivity matrix thresholded at a density level (**e**). Then, a group-based community structure was detected with a community detection algorithm (VTS, IS or GA approach) (**f**). To compare the group-based structure with individual structures, Normalized Mutual Information (NMI) was computed for every pair of the group-based and individual communities (**g**). The individual communities were detected with Louvian algorithm. The obtained NMI values were subjected to the permutation test to determine which algorithm or atlas provided the best representative of the group

For each subject, network graph is constructed from resting-state fMRI data. Then, a single group-based community structure is detected from multiple-subject data and is compared with individual community structures using Normalized Mutual Information (NMI). The obtained NMI values are subjected to a permutation test to determine the algorithm or the atlas that provided the best representative of the group. The processing step is depicted in Fig. 1 and its details are given in the subsequent subsections.

### Subjects

Twenty healthy volunteers (12 female, age: 23.1 ± 3.1 years) took part in the study. All subjects gave written consent to participate in the experiment, and underwent two sessions of fMRI scanning. One out of the two sessions is used and reported in this article. Each subject was reimbursed in compensation for his effort and time. The protocol of the study was approved by the National University of Singapore Institutional Ethical Review Board.

All subjects were right-handed according to the Edinburg Handedness Inventory, and had normal or corrected-to-normal vision. Pre-screening via a short telephone interview was performed to ensure that they had no history of psychological and physical disorders, no poor eyesight, and no metal implants in their body. The subjects were requested to have at least eight hours of sleep for the two nights before the recording, and not to take any caffeine or alcohol for twenty-four hours preceding the study.

### Experimental design

In this study, we used the resting-state functional network to examine agreement of the community structure detected with different community detection techniques or different parcellation techniques. During the resting-state runs, a white fixation cross was presented in the center of the screen, which was projected onto the mirror inside the scanner. The subject was lying calmly on his back with eyes open. The subjects were requested not to move inside the scanner as much as possible. Each run lasted for a period of 5 min and 26 s. Thus, the total number of scans was 163 for each run.

### fMRI data acquisition

All subjects were scanned with a Siemens 3 T Prisma scanner (Siemens, Erlangen, Germany) at the A*STAR-NUS Clinical Imaging Research Centre in the medical school of the National University of Singapore. Functional scans with echo planar imaging (EPI) were acquired using the following parameters: number of slices = 33 (interleaved); slice thickness = 3.5 mm; inter-slice gap = 0.70 mm; matrix size = 74 × 74; flip angle = 90°; repetition time (TR) = 2000 ms; echo time = 30 ms; and in-plane resolution = 3.0 mm. A high-resolution (1.0 × 1.0 × 1.0 mm^3^) sagittal T1-weighted MP-RAGE image was acquired for registration and normalization of functional data to standard brain.

### fMRI data preprocessing

Functional MRI data were preprocessed using FSL (http://fsl.fmrib.ox.ac.uk/fsl/fslwiki/). The functional images were motion corrected, corrected for slice timing, and normalized for intensity. If head motion, which was estimated with the head motion correction algorithm, exceeded 3 mm in translation or 3° in rotation, the motion was regarded as excessive, and the data were to be discarded; however, no data met these criteria. The high-resolution anatomical T1-weighted images were registered to MNI standard space to normalize individual data to the standard space. Average cerebrospinal fluid (CSF) and white matter signals were regressed out with the general linear model (GLM) and the residual signals were used for the subsequent analysis.

### Network construction

To compare different community detection algorithms on multiple-subject data and different brain atlases for parcellation, we constructed functional connectivity maps based on correlation matrices for each subject. Firstly, time series of blood-oxygen-level dependent (BOLD) signal were extracted from the functional data normalized to the standard brain for the regions of interest (ROIs) defined in different brain atlases. The automated anatomical labeling atlas (AAL-90; 90 ROIs) (Tzourio-Mazoyer et al. 2002), Harvard-Oxford probabilistic atlas (HOA; 112 ROIs) (Kennedy et al. 1998; Makris et al. 1999) and Dosenbach atlas (160 ROIs) (Dosenbach et al. 2010) were examined. For HOA atlas, the probability threshold of 25 % was used. The extracted signals were averaged over voxels within each ROI, resulting in 90–160 sets of time-series data, depending on the atlas. The first four volumes of each series were discarded, following which each signal was up-sampled to 1 Hz, band-pass filtered (0.01-0.1 Hz), and down-sampled to 0.5 Hz.

To obtain functional connectivity strength between each node, linear correlation coefficients were calculated for the pre-processed fMRI signals. The correlation coefficients were obtained for all possible pairs of ROIs to construct a correlation matrix *M*. The size of the matrices *M* was different depending on the atlas for parcellation. A Fisher’s-Z transformation was applied to each matrix *M* to improve the normality of the correlation coefficients. As density of connections would influence on partitioning of the network into communities, the obtained functional connectivity matrices were thresholded at density levels of 1–50 % to examine the effects, where higher density level corresponds to denser network. The obtained weighted, undirected graphs were subjected to community detection.

### Community detection based on modularity

Communities or modules are defined as subgroups of densely interconnected nodes in a network (M. E. Newman and Girvan 2004; Radicchi et al. 2004). In this study, we compared different approaches for partitioning networks into communities, i.e., the VTS (Meunier et al. 2009a), the IS (Meunier et al. 2009b) and the GA approaches (Y. Liu et al. 2014). Different brain atlases for parcellation were also compared. All of the examined algorithms for community detection in this study were based on the Louvain algorithm, a fast and relatively accurate algorithm, which maximizes modularity of the community (Blondel et al. 2008). The results of modular decomposition can vary every time the algorithm is executed due to its heuristic nature, but the variation of modularity over the execution was considered to be negligible (Blondel et al. 2008). Therefore, the community detection was performed one hundred times for each connection matrix, and a single modular structure yielding the highest modularity was selected as a representative for subsequent assessment of community structure (Han et al. 2014). Although the highest *Qs* could be observed at several iterations, all community structures showing the highest *Q* were identical for all atlases and all community detection techniques, suggesting reliability of the methods. The community structures were obtained independently at each density level.

For the VTS approach, the community structure of the brain functional network was generated using the averaged connection matrices across subjects (Meunier et al. 2009a; Robinson et al. 2015; Sun et al. 2014). The averaged matrices were thresholded at density levels of 1–50 %. In the Louvain algorithm, the partitioning of the network nodes into communities is completed by maximizing modularity index *Q*, which represents the density of links inside communities compared to links between communities (Blondel et al. 2008), where the definition of modularity index *Q* is given in Appendix 1. For the network with strong community structure, the modularity index typically falls in the range from 0.3 to 0.7 (M. E. Newman and Girvan 2004). The modularity indices and the number of modules were also obtained for fifty different density levels from 1 % to 50 % at the interval of 1 %.

For the IS approach, individual community structures were first detected for all subjects using the Louvain algorithm. Then, Normalized Mutual Information (NMI), which quantifies similarity between two community structures, was calculated for every possible pair of subjects. The detailed calculation about the NMI is given below. The obtained NMI values were averaged for each subject, and the community structure of the subject showing the highest averaged NMI value was selected as the representative (Meunier et al. 2009b).

For the GA approach, a single community structure was detected using individual connection matrices with the algorithm proposed by Liu and colleagues (Y. Liu et al. 2014), an extension of Blondel’s algorithm maximizing the modularity of individual community structures. In this algorithm, the change of modularity was calculated for all subjects excluding outliers, and the change of community structure showing the maximum change of modularity averaged over the subjects is selected at each step of the greedy algorithm to find a community structure showing highest modularity among subjects. In this study, we excluded subjects whose modularity change was outside the 25th and 75th percentiles.

To compare the different community detection algorithms and the different brain atlases for parcellation, we extracted three group-based community structures using three different approaches (VTS, IS and GA) and twenty individual community structures with the Blondel’s algorithm. The group-based and individual community structures were obtained for three different brain atlases (AAL, HOA and Dosenbach atlases).

In-house scripts on MATLAB (version R2011b, Mathworks, USA) were used for network analysis. The flowchart of the fMRI data analysis is depicted in Fig. 1.

### Agreement with individual community structures

**Fig. 2:**
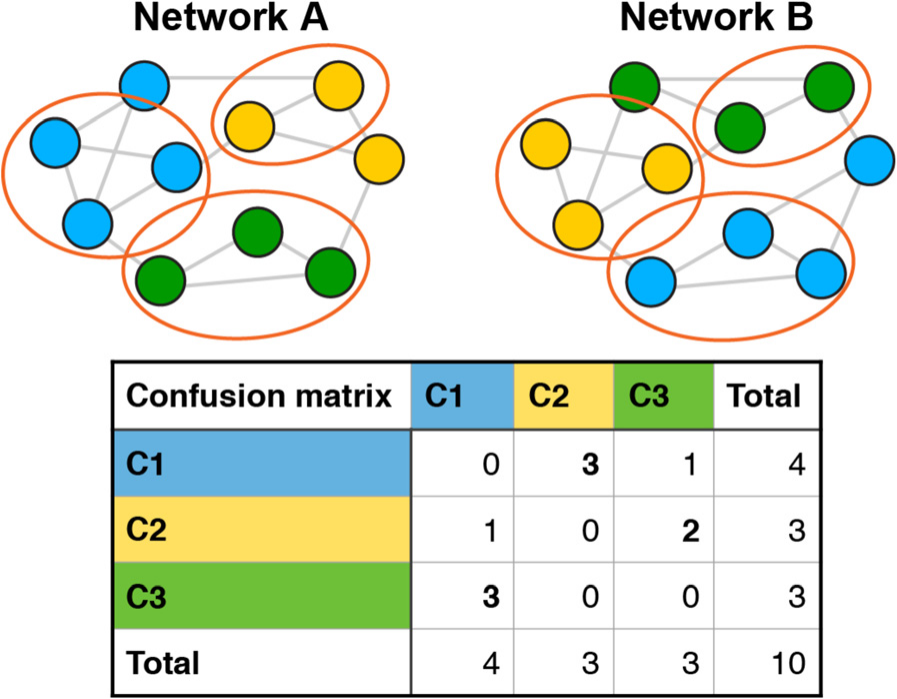
An illustration of calculation of Normalized Mutual Information (NMI) quantifying similarity between two community structures. Colors of the nodes represent assigned community and red circles indicate overlaps in communities. Confusion matrix is used to measure the overlap between the two community structures. In the above figure, Community C1 in Network A corresponds to Community C2 in Network B. The NMI is calculated with the confusion matrix, and gets high when the overlap between the communities is high

To assess the three community detection techniques, using the different brain atlases for parcellation, we propose to examine the similarity between a group-based community and individual community structures with a non-parametric permutation test. The similarity between two community structures is quantified by the Normalized Mutual Information (NMI) (Alexander-Bloch et al. 2012; Kuncheva and Hadjitodorov 2004; Strehl and Ghosh 2003), which is one of the most popular, similarity measure based on information theory. The definition of NMI is given in Appendix 2. The basic idea behind the theory is that, if two communities are similar, less information is necessary to infer one from the other community structure (Fortunato 2010). The confusion matrix, which represents overlap between communities, is useful for understanding the concept of NMI (Kuncheva and Hadjitodorov 2004). If the overlap between communities represented, i.e., the number of nodes belonging to the same community, is high, NMI gets high. The calculation of NMI through confusion matrix is depicted in Fig. 2.

The NMI was calculated for every pair of the group-based community structure (VTS, IS and GA) and the individual community structure (20 subjects) at each density level for three brain atlases (AAL, HOA and Dosenbach). The obtained NMI values were subjected to a non-parametric permutation test in order to examine agreement of group-based community structure with individual community structures. For the comparison of the NMI values, it is examined whether the number of subjects showing higher MNI value for one approach was greater than the other approach. In the permutation test, 10,000 iterations were executed for each density level per subject. To correct for multiple testing, the false discovery rate (FDR) was controlled at the significance level of 5 % (Benjamini and Yekutieli 2001). For comparison, a permutation test on averaged NMI values was also performed.

### Permutation tests on modularity indices

To compare the modularity index of the intrinsic functional network for different approaches, we used a permutation test with 10,000 iterations for each density level per subject. For modularity indices, we recalculated the modularity indices using the group-based community structure detected with VTS, IS and GA approaches and the individual connection matrices. Please note that the modularity indices used for the statistical tests were different from those used during the community detection. To correct for multiple testing, the false discovery rate (FDR) was controlled at the significance level of 5 % (Benjamini and Yekutieli 2001).

### Consistency in community structures within iterations

To overcome the heuristic nature of the algorithms for community detection, the partition of the network was performed 100 times and a community showing the highest modularity was selected as a representative. However, this approach can be applied only to networks with relatively small number of nodes (90–160 parcellated regions in this study). For larger networks, it is too time-consuming to repeat the calculation multiple times.

In order to grasp the nature of the community detection algorithms, the obtained 100 community structures were investigated. For each atlas, group-based communities detected with VTS and GA algorithms were compared. At each density level, NMI values between a group-based community and 20 individual communities were calculated for 100 communities. The obtained NMI values were first averaged over subjects. Then, the averaged NMI values across iterations and standard deviation of the values were calculated to investigate consistency in community structure within iterations in terms of agreement with individual community structures. Additionally, in order to explore similarity between detected community structures within 100 iterations, NMI values were calculated for all possible pairs of the detected 100 communities.

## Results

### Modularity and the number of communities

**Fig. 3:**
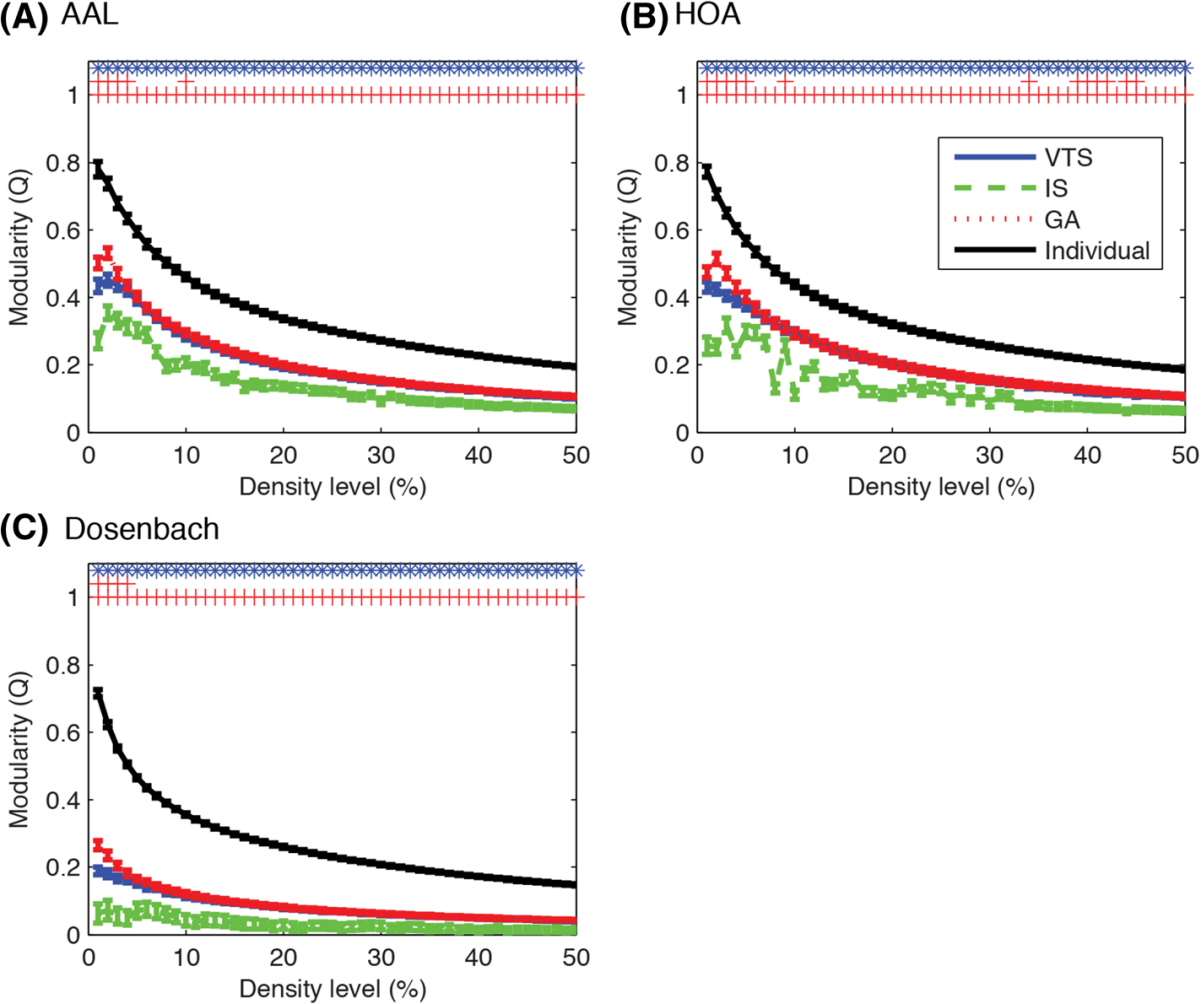
Averaged modularity indices *Q*s were better for VTS and GA, compared with IS approach. Three rows of symbols on the top of each figure show significant difference (*p* < 0.05, FDR-corrected) for the comparison of VTS-IS, VTS-GA and IS-GA. Blue asterisks, green Xs and red crosses indicate significant higher *Q* values for VTS, IS and GA respectively. Error bars indicate SEMs

**Fig. 4:**
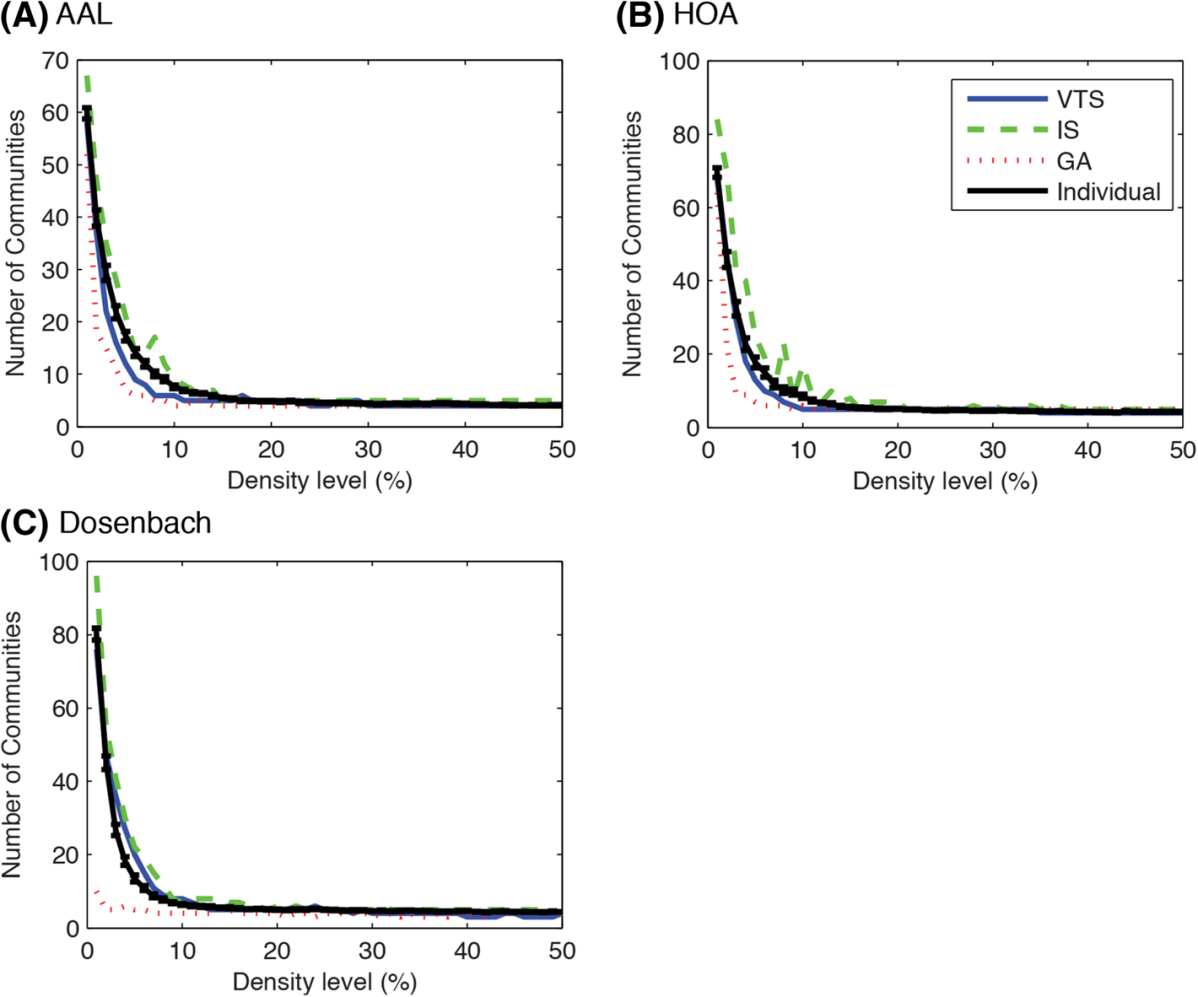
IS approach tended to identify more communities, while GA approach detected the least. Error bars indicate SEMs

We first compared the global properties of the community structure: the modularity *Q* and the number of communities. Figure 3 shows the recalculated modularity indices averaged over subjects while Fig. 4 shows the number of communities of the group-based community structures. The modularity indices and the number of communities for individual community structures are also shown in these figures. As can be seen in Figs. 3 and 4, the modularity indices and the number of communities decreased as the density level increased. Generally, the averaged modularity indices were higher for VTS and GA compared to IS approach while VTS and GA approaches produced the group-based communities with almost the same modularity at every density level in all atlases. As expected, the averaged modularity index of individual community structure was highest for all atlases as Louvian algorithm looks for the community structure showing the highest modularity. For the number of communities, IS approach tended to detect more communities while GA approach tended to detect fewer communities. VTS approach lay in the middle.

Permutation tests on modularity indices showed that both VTS and GA approaches communities with higher modularity indices compared to IS approach at every density level. When comparing VTS and GA, GA approach produced community with higher modularity at a number of density levels (AAL: 1–4, 10 %, HOA: 1–5, 9, 34, 39–42, 44–45 %, Dosenbach: 1–4 %) while no community detected with VTS approach showed higher modularity than GA.

### Agreement with individual community structures

**Fig. 5:**
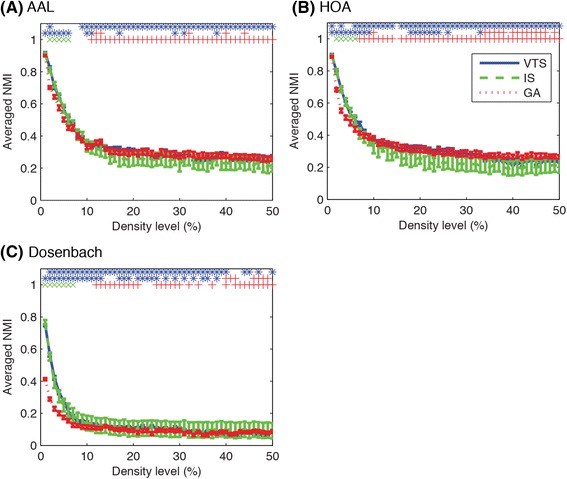
Averaged NMI between group-based consensus community structure and individual community structures are shown for different community detection methods. VTS and GA approaches identified better consensus community structures in terms of the agreement of group-based community structure with individual community structures. Three rows of symbols on the top of each figure show significant difference (*p* < 0.05, FDR-corrected) for the comparison of VTS-IS, VTS-GA and IS-GA, using the proposed method. Blue asterisks, green Xs and red crosses indicate significant higher NMI values for VTS, IS and GA respectively. Error bars indicate SEMs

**Fig. 6:**
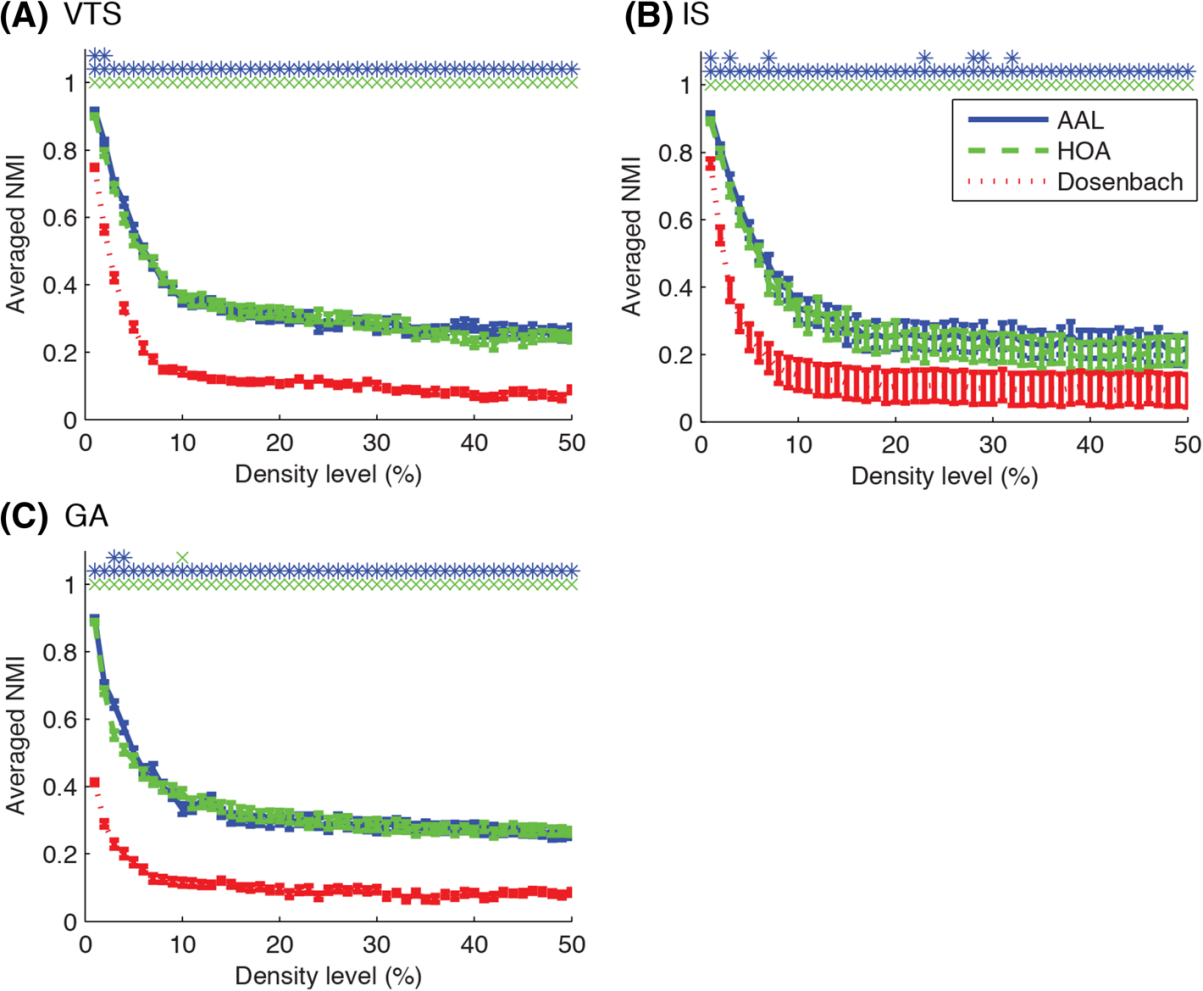
Averaged NMI between group-based consensus community structure and individual community structures are plotted for different brain atlases. AAL and HOA atlases tended to produce better consensus community structures, compared to Dosenbach atlas in terms of the agreement of group-based community structures with individual community structures. Three rows of symbols on the top of each figure show significant difference (*p* < 0.05, FDR-corrected) for the comparison of AAL-HOA, AAL-Dosenbach and HOA-Dosenbach. Blue asterisks, green Xs and red crosses indicate significantly higher NMI values for AAL, HOA and Dosenbach atlas respectively. Error bars indicate SEMs

To compare different community detection algorithms and brain atlases for parcellation, we examined the agreement of the group-based community structure with the individual community structures. To quantify the similarity of the group-based community structure with the individual community structures, we computed NMI values between the group-based and the individual community structures for each approach. Figure 5 shows the averaged NMI values between the group-based community, partitioned with different community detection techniques, and individual communities for each brain atlas while Fig. 6 shows the averaged NMI values between the group-based community for different brain atlases and individual communities plotted for each community detection approach. As shown in these figures, the agreement with individual community structures decreased as the density level increased, suggesting that it is harder to find a single common community structure as the density of the network increases.

When comparing the different community detection approaches, a small difference existed in averaged NMI values between them. The GA approach tended to show lower NMI values for sparser networks, as shown in Fig. 5. In order to get a clearer picture of which method was superior to the other, we carried out a non-parametric permutation test on the number of subjects showing higher NMI value between the compared methods. The permutation test demonstrated significant differences at a number of density levels. In comparison to IS, VTS produced communities with greater number of subjects showing higher NMI values at density levels of 2, 9–50 % in AAL, 1, 3, 6–14, 15–50 % in HOA, and 2–40, 44–45, 47, 50 % in Dosenbach atlases. When comparing VTS with GA, VTS tended to show higher agreement with individual community structures at lower density levels (AAL: 1–6, 10–11, 17, 29, 31, 38 %; HOA: 1–9, 18–24, 26, 33 %; Dosenbach: 1–13, 17–19, 21–25, 27, 29, 31, 33, 35–36, 38 %) while GA tended to show higher agreement at higher density levels (AAL: 12–13, 32, 40, 44 %; HOA: 10, 34–38, 40–47, 49 %; Dosenbach: 40–42, 46–49 %). In the comparison of IS with GA, IS tended to show higher agreement at lower density levels (AAL: 2–6 %; HOA: 2–6 %; Dosenbach: 1–7 %) while GA tended to show higher agreement at higher density levels (AAL: 11–50 %; HOA: 7–14, 16–50 %; Dosenbach: 12–21, 25–30, 32, 34, 37, 40, 42–50 %).

The proposed test can be applied for the comparison between brain atlases used for parcellation. As shown in Fig. 6, Dosenbach atlas showed lower agreement with individual community structures than both AAL and HOA at every density level, which was further confirmed with the permutation tests (*p* < 0.05, FDR-corrected). In comparison of AAL with HOA, AAL showed higher agreement at a number of density levels (VTS: 1–2 %; IS: 1, 3, 7, 23, 28–29, 32 %; GA: 3–4 %) while HOA showed higher agreement at a density level of 10 % for GA.

When averaged NMI values were tested, the permutation test was less sensitive compared to that on the number of subjects. For example, VTS-IS comparisons showed no significant difference for AAL and Dosenbach atlases probably due to large variability in the IS algorithm. Even for the other comparisons, the permutation tests on averaged NMI values showed less sensitivity to the difference between algorithms or atlases in general. These results indicate that the permutation test on the averaged NMI values is more sensitive to outliers.

### Similarity between group-based community structures

**Fig. 7:**
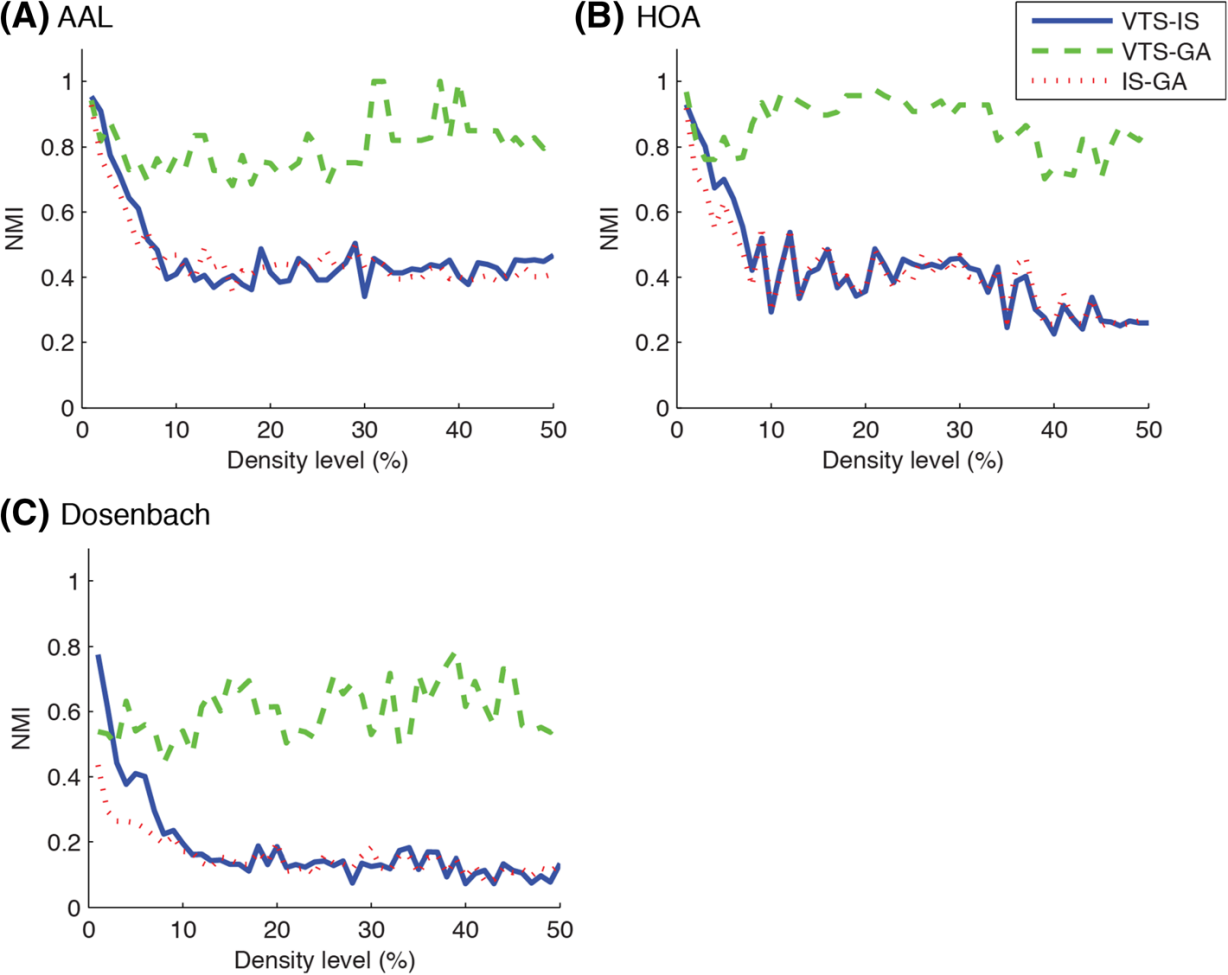
NMI between group-based community structures detected with different approaches are shown. VTS and GA approaches tended to produce similar group-based community structures regardless of brain atlases, while IS approach identified quite different ones

To investigate similarities between group-based community structures detected with different community detection algorithms, NMI values between group-based communities were calculated as shown in Fig. 7. As can be seen in the figure, NMI values between VTS and GA approaches fluctuated around relatively higher values (0.8–0.9) suggesting that the VTS and the GA tended to produce relatively similar community structures. On the other hand, NMI for VTS-IS and IS-GA were very low particularly for higher density levels, suggesting that the IS approach produced very different community structures from the other approaches.

### Consistency in community structures within iterations

**Fig. 8:**
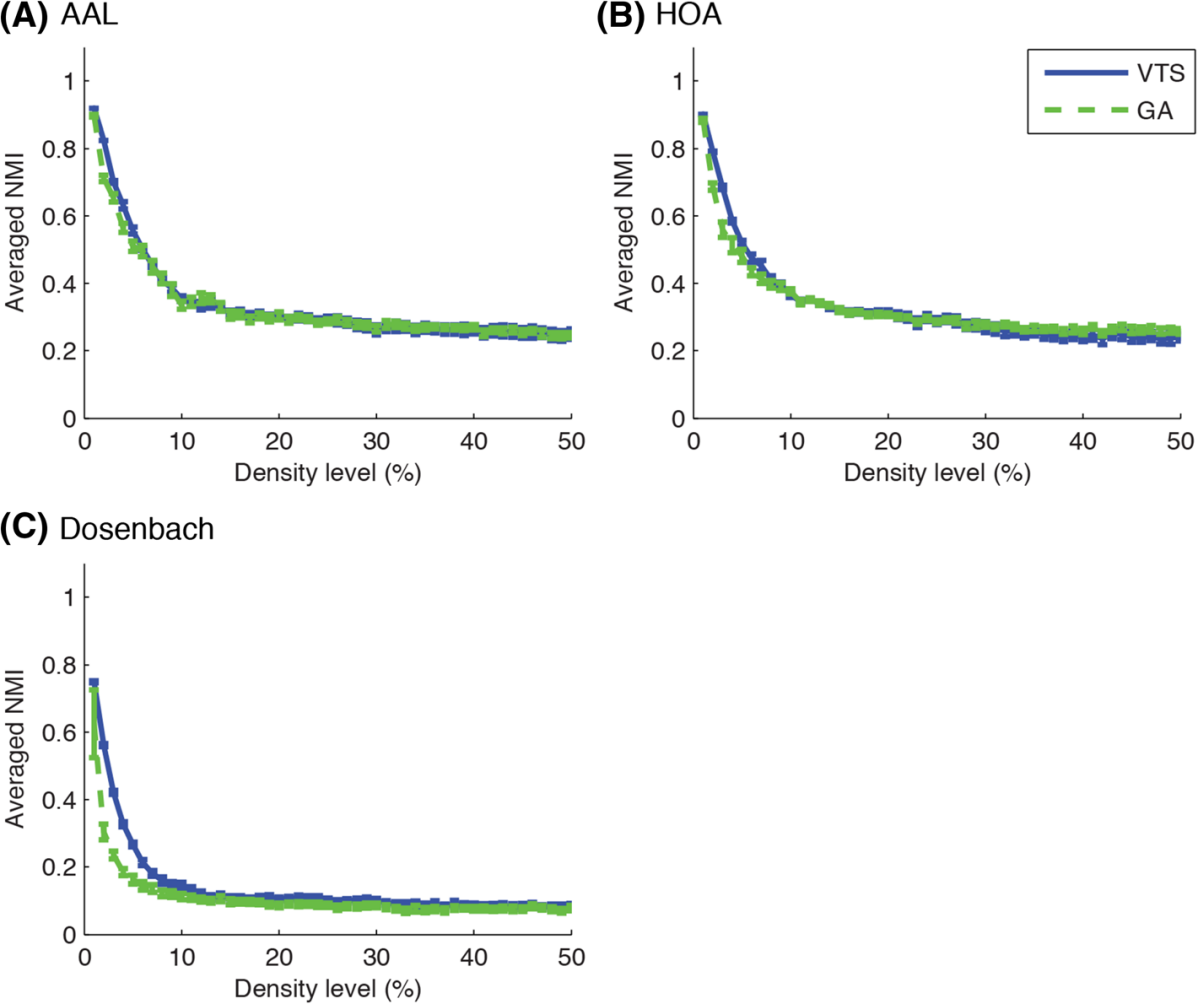
Averages and standard deviations of NMI between group-based and individual community structures over 100 iterations of community detection are shown. Variability in the agreement of the obtained consensus communities with the individual structures was very small in both approaches

To investigate consistency in community structures within iterations of community detection, the averaged NMI values and the standard deviations of the values over iterations were represented in Fig. 8. As seen in the figure, the variability of the averaged NMI values over iterations was very small at every density level for both VTS and GA regardless of the brain atlas. These results indicate that both community detection algorithms produce consistent communities at every iteration in terms of agreement with individual community structures, suggesting that even a single execution of the community detection can produce a group-based community with comparable quality.

**Fig. 9:**
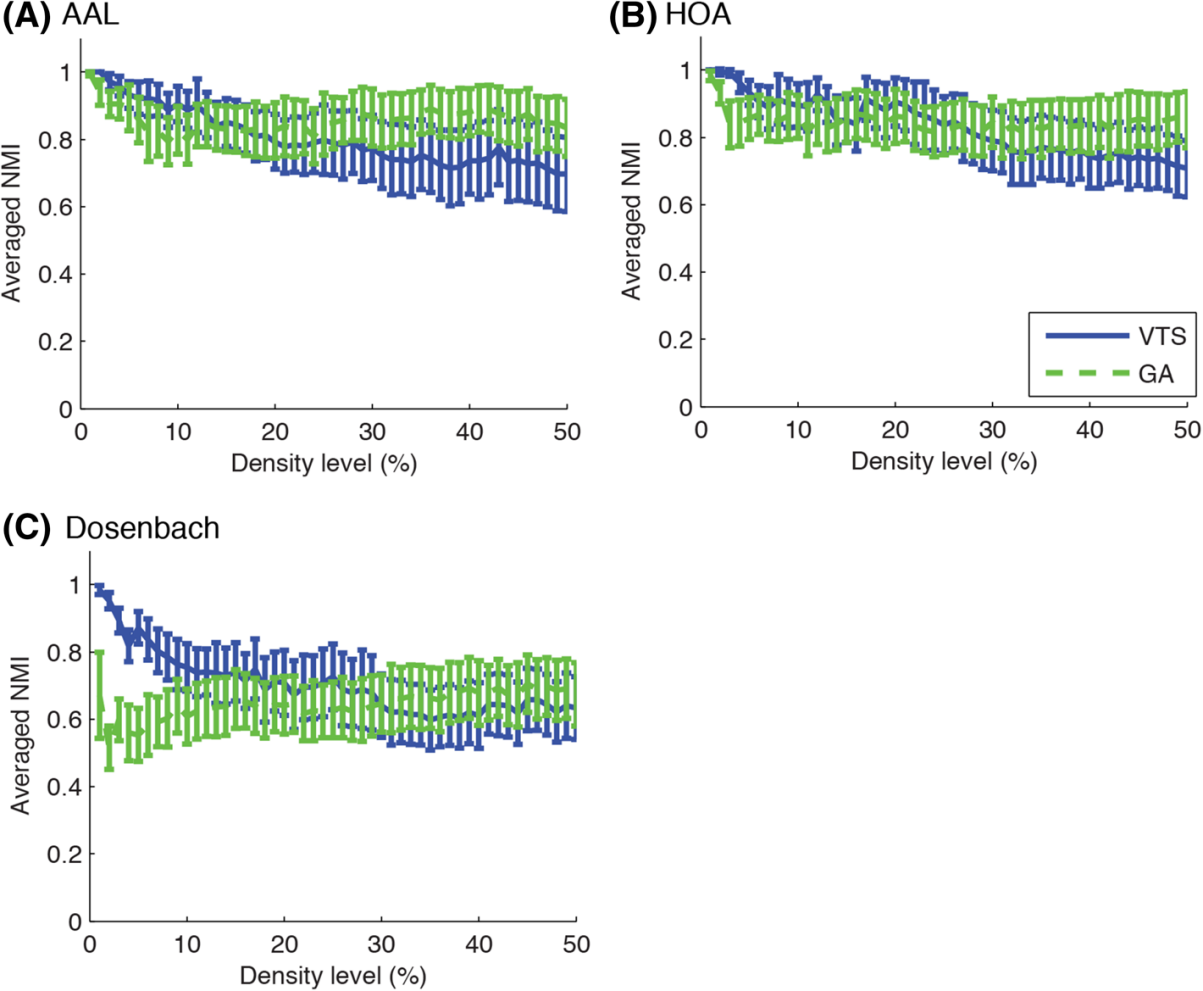
Averages of NMI between every pair of obtained community structures within 100 iterations are shown. VTS approach tended to identify similar group-based communities at lower density levels while GA tended to produce similar group-based communities at higher density levels. Error bars indicate SDs

Similarity of the obtained community structures within 100 iterations was also explored as shown in Fig. 9. Generally, VTS produced communities with higher resemblance for sparser networks, but the similarity became lower for denser networks. On the other hand, GA produced relatively consistent results at every density level, except at lower density levels for Dosenbach atlas.

### Summary

In summary, comparing between community detection algorithms, the VTS and the GA approaches detected community structures with greater agreement with individual community structures, as compared to the IS approach (Fig. 5). When comparing the representative group-based communities detected with the different algorithms, the VTS and the GA approaches produced relatively similar results while the IS approach detected very different community structures particularly at higher density levels (Fig. 7). Additionally, the VTS approach tended to produce better results at relatively lower density levels (Fig. 8) while the GA approach produced stable results regardless of the density levels (Fig. 9). When comparing brain atlases for used parcellation, AAL and HOA atlases were comparable while Dosenbach atlas produced surprisingly worse results than the other atlases (Fig. 6).

## Discussion

In this study, we proposed the method to compare different schemes for community detection from multiple-subject neuroimaging data to determine which algorithm or atlas produce the best representative community structure of the group. To the best of our knowledge, this is the first attempt to assess community structure schemes for multiple-subject data.

We showed that our method was feasible to real data: brain functional networks derived from resting-state fMRI data. For this purpose, three community detection algorithms based on different approaches (i.e., VTS, IS and GA), and three brain atlases (i.e., AAL, HOA and Dosenbach atlases) used in parcellation were examined. In our method, the Normalized Mutual Information (NMI) was calculated between a group-based community and each individual community. The obtained NMI values were subjected to the permutation test to examine whether there was a significant difference in the number of subjects showing a greater NMI value for either of the approaches examined. Our method showed significant differences between community detection schemes even when no significant difference was found on averaged NMI, suggesting that our method was more sensitive to the difference in NMI values between algorithms or those between atlases.

Our results indicate that the VTS approach, using the averaged connection matrix as the representative, can produce surprisingly good results. For the VTS approach, group-based connectivity pattern can be obtained using more sophisticated method rather than just averaging such as the modified PC_FDR_ algorithm based on a multi-subject, error-rate-controlled brain connectivity modeling approach (Liu et al. 2012), which could result in better results. As the GA and the IS approaches are computationally more expensive, the VTS approach proved to be a relatively good choice. However, please note that our results are limited to the particular algorithms and dataset that we examined. Although it is inconsistent with the result showing higher accuracy of the GA approach in community detection compared to the VTS approach for EEG simulated data (Y. Liu et al. 2014), there are several potential reasons for this, e.g., difference in data modality (fMRI vs. EEG), temporal resolution of signals and spatial distribution of sensors. Also, a more elaborate version of the IS approach could produce communities with higher agreement with individual structures. Although, in the IS algorithm that we examined, a community showing the highest average NMI value was selected as a representative, other algorithms following the IS approach can be implemented by integrating individual structures with averaging or voting/consensus algorithms (Strehl and Ghosh 2003).

In the Dosenbach atlas that showed the worst result, 160 ROIs are identified functionally and are defined as spheres centered at peaks identified with meta-analyses (Dosenbach et al. 2010) while the ROIs are defined anatomically in AAL and HOA atlases. Therefore, the functional distinctiveness of ROIs may be too prominent in Dosenbach atlas and thus resulted in poorer clustering of ROIs in community detection. Other community detection algorithms and brain atlases are to be investigated.

In addition to the quality of the obtained common community, the scalability of different methods must be considered to determine the scheme for community structure analysis on neuroimaging data. First, although we used a greedy Louvain algorithm, which tries to maximize modularity, for community detection, there are a variety of algorithms based on different approximation methods as the community detection or clustering is NP-hard (Fortunato 2010). Second, the VTS, the IS and the GA approach are also different in scalability. Generally, the VTS approach requires the least computation as the community detection is performed on an averaged network. Thus, it depends only on the community detection algorithm. The IS approach requires to obtain individual community structures, indicating that the computational time depends on the number of individual networks. However, if the individual communities are already given or necessary for other purposes, the relative costs could be comparable with the VTS approach. The GA approach lies between the VTS and the IS approaches. Furthermore, the network size, i.e., the number of nodes, is crucial although the computational time could be less important compared with social or biological networks whose network size is usually much larger than that of brain networks. In case of fMRI brain network, the number of nodes depends on the choice of brain atlas. Practically, the computational time is almost negligible for the AAL atlas whose node number is 90, but much longer for the Dosenbach atlas or other atlases such as Cradock atlas consisting of 200 ROIs (Craddock et al. 2012).

There are further several issues to be addressed for community detection on neuroimaging data. Although the community detection is a promising approach for studying mesoscale organization of brain network because of its well-established modular architecture, there are no established schemes for the employment of the community detection analysis on neuroimaging data obtained from multiple subjects. Currently, there are two approaches to investigate the modular organization of the brain network. Firstly, a single group-based community structure is obtained with a community detection technique based on any one of the virtual-typical-subject (VTS), the individual structure (IS), or the group analysis (GA) approach because a single community structure common among individual subjects is necessary to discuss about brain regions and connections on common ground. Secondary, quantitative metrics measuring global properties of community structure, such as modularity index or the number of communities, are computed based on individual community structure and discussed (Bassett et al. 2011). In either case, the community structure analysis is not used effectively, demanding more sophisticated approaches to neuroimaging data. In addition, although there are several options for partitioning brain networks into communities and for dividing whole brain into regions or nodes, there exists no established scheme for obtaining community structures. Further studies would be necessary to investigate effects of different scheme on community structure analysis.

In this study, we proposed a method using the permutation test on Normalized Mutual Information to compare different community detection approaches to neuroimaging data from multiple subjects. Using the real fMRI data, we demonstrated that our method is feasible for comparing different community detection algorithms and different brain atlases for parcellation. The results showed that the algorithms based on VTS and GA approaches are comparable and detected a group-based community structure showing greater agreement with the individual community structures compared to IS approach, and anatomically defined AAL and HOA atlases are more appropriate than functionally defined Dosenbach atlas for community structure analysis. Our method would be a useful tool to evaluate different approaches for detecting a group-based community structure from multiple-subject data in terms of the agreement with individual community structures.

## Availability of data and materials

The brain network data used in this study are available at "http://sinapseinstitute.org/projects/cognitiveengr/network_data/". Please refer to Methods in this article for the details about the datasets.

## Appendix 1

### Modularity

In this study, we used Louvain algorithm, which is a greedy method trying to optimize a quality function of clusters, to identify community structures of the brain network (Blondel et al. 2008). The most popular quality function is the modularity of Newman and Girvan (M. E. Newman and Girvan 2004), which was also used in our study. The modularity index *Q* for weighted networks is defined as (Blondel et al. 2008):

$$ Q=\frac{1}{2m}{\displaystyle \sum_{i,j}\left[{W}_{ij}-\frac{k_i{k}_j}{2m}\right]}\delta \left({c}_i,{c}_j\right) $$

where *W*_*ij*_ is the weight of the edge between the node *i* and the node *j*, *k*_*i*_ is the sum of the weights of the edges linking to the node *i*, *c*_*i*_ is the community to which the node *i* belongs to, the *δ*-function *δ(u,v)* is 1 if *u* = *v* and 0 otherwise, and *m* = ∑_*i*,*j*_ *W*_*ij*_. If the sum of the weights of the edges within the communities is no more than that of random network, *Q* will be 0. *Q* approaches to 1 if the network has a strong community structure.

## Appendix 2

### Normalized mutual information (NMI)

To measure similarity between community structures, Normalized Mutual Information (NMI), which is one of the most popular metrics for comparing communities, is used (Fortunato 2010; Strehl and Ghosh 2003). NMI is defined as follows.

$$ NMI\left(A,B\right)=\frac{-2{\displaystyle \sum_{i=1}^{C_A}{\displaystyle \sum_{j=1}^{C_B}{N}_{ij} \log \left(\frac{N_{ij}N}{N_{i.}{N}_{.j}}\right)}}}{{\displaystyle \sum_{i=1}^{C_A}{N}_{i.} \log \left(\frac{N_{i.}}{N}\right)}+{\displaystyle \sum_{j=1}^{C_B}{N}_{.j} \log \left(\frac{N_{.j}}{N}\right)}} $$

Where *A* and *B* are the community structure of two graphs; *C*_*A*_ is the number of communities in partition *A*; *C*_*B*_ is the number of communities in partition *B*; *N* is the number of nodes, which is the same in both community structures; *N*_*ij*_ is the overlap between *A*’s community *i* and *B*’s community *j*, i.e. the number of nodes that the communities have in common; *N*_*i.*_ is the total number of nodes in *A*’s community *i*; *N*_*.j*_ is the total number of nodes in *B*’s community *j*; and this calculation follows the convention that 0 × log(0) = 0. The NMI ranges from 0 to 1, where 0 signifies that the community structures are totally independent and 1 that they are identical.

## Abbreviations

AAL: automated anatomical labeling
BOLD: blood-oxygen-level dependent
CSF: cerebrospinal fluid
EEG: electroencephalography
EPI: echo planar imaging
FDR: false discovery rate
fMRI: functional Magnetic Resonance Imaging
GA: group analysis
GLM: general linear model
HOA: Harvard-Oxford Atlas
IS: individual structure
NMI: Normalized Mutual Information
ROI: region of interest
VTS: virtual-typical subject
